# Engagement with Health Agencies on Twitter

**DOI:** 10.1371/journal.pone.0112235

**Published:** 2014-11-07

**Authors:** Sanmitra Bhattacharya, Padmini Srinivasan, Phil Polgreen

**Affiliations:** 1 Department of Computer Science, The University of Iowa, Iowa City, Iowa, United States of America; 2 Department of Internal Medicine, The University of Iowa, Iowa City, Iowa, United States of America; Rollins School of Public Health, Emory University, United States of America

## Abstract

**Objective:**

To investigate factors associated with engagement of U.S. Federal Health Agencies via Twitter. Our specific goals are to study factors related to a) numbers of retweets, b) time between the agency tweet and first retweet and c) time between the agency tweet and last retweet.

**Methods:**

We collect 164,104 tweets from 25 Federal Health Agencies and their 130 accounts. We use negative binomial hurdle regression models and Cox proportional hazards models to explore the influence of 26 factors on agency engagement. Account features include network centrality, tweet count, numbers of friends, followers, and favorites. Tweet features include age, the use of hashtags, user-mentions, URLs, sentiment measured using Sentistrength, and tweet content represented by fifteen semantic groups.

**Results:**

A third of the tweets (53,556) had zero retweets. Less than 1% (613) had more than 100 retweets (mean  = 284). The hurdle analysis shows that hashtags, URLs and user-mentions are positively associated with retweets; sentiment has no association with retweets; and tweet count has a negative association with retweets. Almost all semantic groups, except for geographic areas, occupations and organizations, are positively associated with retweeting. The survival analyses indicate that engagement is positively associated with tweet age and the follower count.

**Conclusions:**

Some of the factors associated with higher levels of Twitter engagement cannot be changed by the agencies, but others can be modified (e.g., use of hashtags, URLs). Our findings provide the background for future controlled experiments to increase public health engagement via Twitter.

## Introduction

Government agencies are increasingly interested in using social media to distribute information at the national, state and local levels. U.S federal agencies, for example, routinely use a variety of social media sites including Twitter, Facebook, YouTube, Flickr, and Instagram to enhance communication [1]. In addition to distributing information, government agencies are increasingly interested in interacting with the populations they serve. For example, new guidelines entitled “Digital Governmental Strategy” outline specific steps for governmental agencies to make digital information more “customer centric” [2]. This bidirectional form of communication can be defined as engagement: interactions designed to promote some common goal [3].

To date no study has systematically explored factors associated with the levels of health agency engagement on social media. Our objective is to address this gap by using retweeting as a measure of engagement. Specifically we address the following three questions with respect to Twitter messages posted by US Federal Health agencies and their responses. First, which features are associated with the level of response in the form of retweets? Second, which features are associated with the interval between an agency's tweet and its first retweet? Third, which features are associated with the interval between an agency's tweet and the last retweet it generates? We address our goals by analyzing an almost comprehensive set of tweets posted by the 130 Twitter accounts of 25 Federal Health Agencies. We explore associations between factors with level of retweeting using hurdle models. We explore the temporal factors related to our second and third questions using survival models. Factors we examine include standard features such as the number of friends and followers as well as less studied features relating to the semantic content of a tweet.

## Background and Significance

The U.S. government uses several social media services, but Twitter is one of the most commonly used service. Recent estimates indicate that approximately 18% of online adults use Twitter [4], and over 500 million users around the globe [5] generate over 500 million tweets per day [6]. Given the widespread use of Twitter and the fact that people are increasingly using it to share their experiences with illness and treatments as well as other health concerns [7], Twitter provides a potentially valuable stream of health-related information. Several studies have used Twitter to discover adverse drug reactions [8], [9], perform surveillance for disease activity [10], [11] and health beliefs [12], [13]. Twitter has also been used to investigate general health behavior [14], [15]. However, few studies have focused on how health agencies use Twitter. The studies that do exist describe activity consistent with distributing information with little attention paid to engagement [16]. One of the few studies on engagement via Twitter focuses on levels of engagement: low (have followers), medium (promote retweeting) and high (have offline interactions) [3]. In contrast, to previous studies, our goal is to determine the *factors* associated with engagement of federal agencies with the “Twitter Public”. The caveat to note is that while we focus on public engagement an agency may be equally or even more interested in information dissemination alone.

We study factors related to engagement in terms of retweeting activity. A retweet is an acknowledgment that the original tweet has been read and also that it is viewed as sufficiently interesting to merit a re-post. The followers of the retweeting account now have access to the original retweet. Retweets are in some sense analogous to citations in an article. A second aspect of engagement relates to the time period over which retweeting occurs. A tweet with a longer retweeting time span compared to another is one where engagement occurs over a longer period of time. Thus, Twitter engagement for a federal agency is maximized when all of its tweets generate the highest possible number of retweets with retweets starting almost immediately after the tweet is posted and continuing on forever. While in practice these conditions are never achieved, it is clear that some tweets generate stronger responses than others. Our overarching goal is to determine whether there are features that relate to higher levels of retweeting and longer lifespans of tweets in order to offer insight into ways to gauge and strengthen Twitter engagement for health agencies.

## Methods

### Data Collection

#### Agencies & Handles

We selected health agencies through the HHS Social website, which maintains a list of all official HHS-affiliated accounts across various social media platforms [17]. We identified all agencies with Twitter accounts (also known as handles).

#### Tweets & Retweets

The Twitter REST API v1.1 [18] was used to collect all tweets from a handle's timeline as of late November 2012 (data collection was done between 11/20/2012-11/21/2012). Using this method, a maximum of 3200 tweets from a handle's timeline can be retrieved. These timelines extended from a few months (e.g., around 3 months for CDCSTD) to several years (e.g., around 3 years for NIGMS). On average the timeline was around 2 years for all handles. We could collect all posted tweets for 112 handles; 18 handles had more than 3200 tweets at the time of data collection so the data for these handles was censored. The average timeline for these handles also spanned around 2 years. Handles such as CDCSTD, womenshealth and CDCNPIN had posted over 9000 tweets by the time of the data collection. For such handles the most recent 3200 tweets were collected. For each agency tweet, we recorded its unique identifier and raw retweet count among other tweet-based data and metadata as described below.

### Tweet Features

First we decided which features we would use to represent each tweet. We included those examined commonly in Twitter-based studies as well as those that have not yet been considered. Table 1 lists 11 features we considered under 2 broad categories: handle-level features that are the same for all tweets issued by a handle (e.g., numbers of followers and friends) and tweet-specific features such as sentiment.

**Table 1:** Features Examined.

Type	Group	Features	Description
Handle-level	1	Favorites	# of users favoriting tweets of a particular handle (log-transformed).
	1	Followers	# of users following a particular handle (log-transformed).
	1	Friends	# of users followed by a particular handle (log-transformed).
	1	Betweenness-centrality	Importance of node in network.
	2	Tweet count	# of tweets posted by a handle in its lifetime (log-transformed).
Tweet-level	1	Tweet age	# of days between handle creation and tweet post (log-transformed).
	2	Hashtag	Whether a tweet contains a hashtag, word prefixed with # (binary).
	2	URL	Whether a tweet contains an URL, http, ftp, etc. (binary).
	2	User-mention	Whether a tweet contains a user-mention, word prefixed with @ (binary).
	2	Sentiment	Two scores: one for positivity and another for negativity.
	2	Content (Semantic Groups)	Classification of each tweet into 15 semantic groups using MTI followed by post-processing. Multiple classes per tweet allowed.

We also divided the features into two logical groups. Group 1 has features that cannot be changed or easily manipulated by an account holder. We include tweet age in this group as it represents a natural phenomenon. The account holder has control over Group 2 features.

Group 1 features include the number of followers, friends and favorites. If user Y is a follower of user X then it means that Y receives all of X's tweets automatically. Also, X is regarded as a friend of Y. Relevant to us is that a tweet is displayed on the timelines of all of its handle's followers, so these are the users most likely to retweet the post. The feature favorite is the number of users favoring a particular handle. Twitter forms a network due to its follower and friend relationships between users. From this network, we calculate a betweenness-centrality score. This shows the extent to which a node acts as an intermediary in the shortest paths between nodes in the network; it indicates the importance of a particular node in the network structure.

We analyzed sentiment using a state-of-the-art lexicon-based sentiment classifier, SentiStrength [19], [20]. SentiStrength has been widely applied for sentiment analysis of tweets [21] and has been shown to outperform other lexical classifiers [22]. SentiStrength classifies each tweet into positive and negative sentiments on a scale of +/−1 (neutral) to +/−5 (extreme).

One aspect of tweet analysis that is often overlooked in Twitter studies is the content of the tweets. The exception is in the few studies focused on specific domains (e.g., manual coding of 1,000 concussion-related tweets along 9 broad themes [23]). Content is important as some subjects may attract a broader audience than others. In order to analyze tweet content, we design a fully automated method for content analysis. Manual analysis is not feasible as it limits the number of tweets that can be content coded. We use the National Library of Medicine's Medical Text Indexer (MTI) [24], [25] to assign Medical Subject Headings (MeSH) [26], [27] recommendations for each tweet. MTI is commonly used for recommending MeSH terms to biomedical literature based on the titles and abstracts. It has been shown to be useful in other domains such as clinical text [25]. The terms recommended for each tweet are mapped into semantic types [28], which in turn are assigned to semantic groups [29], [30]. Note that a particular tweet can be assigned to multiple semantic groups.

### Choice of Models

The number of retweets per tweet in our dataset is highly skewed with many zeros. This type of data distribution where the variance is much greater than the mean is described as overdispersed [31] with zero-inflation [32]. Typically models such as Poisson or negative binomial regression are used to model count data. However the zero-inflation of the retweet count necessitates the use of two-part count data models such as the hurdle regression model [33]–[35].

Hurdle models have two separate components: a zero-portion to model the inflation of zero counts in the data and a count-portion to model the non-zero counts of the data. The zero-portion determines the binary outcome of whether a count is zero (no retweets) or not using a binomial probability model. The count portion of the model determines the conditional distribution of the non-zero count of the data using a zero-truncated negative binomial or Poisson model.

We formally compare different count data regression models (namely, the Poisson (P), negative binomial (NB), hurdle Poisson (HP) and hurdle negative binomial (HNB)) using standard goodness-of-fit measures [36], [37]. The Poisson model is the hurdle Poisson model with the zero component ignored. Thus the Poisson model is ‘nested’ in the ‘full’ hurdle Poisson. Similarly the negative binomial is nested in the hurdle negative binomial model. Akaike information criterion (AIC) and Vuong statistics are used to compute goodness-of-fit for all pairs of nested and full models (e.g. NB vs. HP, HNB vs. NB, etc.). The HNB model had the lowest AIC value (800270.2) compared to the Poisson (2649779), negative binomial (813296.6) and hurdle Poisson (2274348) models, signifying a better fit. The Vuong statistics for hurdle negative binomial compared to Poisson, negative binomial and hurdle Poisson were 73.89, 14.43 and 59.36 respectively, all significant at p<0.001. This signifies that hurdle negative binomial model has a better fit compared to the other models. Our comparison of full and nested models such as hurdle negative binomial and negative binomial using the likelihood ratio test (LRT) also corroborates to other goodness-of-fit measures in implying that the former model fits our data best.

In addition, we use methods from survival analysis [38], [39], to model the temporal aspects of retweeting. Typically in survival analysis we build models to analyze “time to events” such as death of an organism or failure of a machine [40]. In our case, we estimate two survival models. For the first model, the “event” refers to the time until the appearance of the first retweet. For the second model, the “event” is the time to the last retweet of a tweet – the length of time that the tweet is in “circulation”. Similar to previous Twitter research [41] we use the Cox proportional hazards regression model [42] to predict how the different handle and tweet-based features influence the time to the first and last retweets.

## Results

### Tweets

A total of 134 Twitter accounts were identified out of which 4 were either deleted or suspended or had no tweets posted in their lifetime. We used the remaining 130 handles in our study. These correspond to 25 different health agencies that include ACF, AHRQ, CDC, CMS, FDA, HRSA, NIH, OIG, OS, SAMHSA and fifteen NIH subdivisions (NIA, NCCAM, NCI, NEI, NHLBI, NIAAA, NIAID, NIAIMS, NICRR, NIDA, NIEHS, NIGMS, NIHGRI, NIMH, NLM). Some agencies have quite a few handles such as NIH/NCI (13 handles: SmokefreeGove, NCIHINTS etc.), CDC (25 handles: CDCgov, CDCActEarly etc.), FDA (10 handles: US_FDA, FDATobacco etc.), while others have just one handle such as AHRQ, ACF and NIH/NEI. Table 2 lists the various agencies (including their expanded names), the number of handles for each and a few examples of handles.

**Table 2:** Agencies and Handles.

Agency	Name	# handles	Examples of handles
ACF	Administration for Children & Families	1	HeadStartgov
AHRQ	Agency for Healthcare Research & Quality	1	AHRQNews
CDC	Center for Disease Control & Prevention	25	CDCgov, CDCActEarly, CDC_BioSense, etc.
CMS	Centers for Medicare & Medicaid Services	4	CMSGov, CMSinnovates, IKNGov, etc.
FDA	U.S. Food & Drug Administration	10	US_FDA, FDATobacco, FDADeviceInfo, etc.
HRSA	Health Resources & Services Administration	1	HRSAgov
NIH	National Institutes of Health	15	NIHforFunding, NIHClinicalCntr, etc.
NIH/NIA	National Institute on Aging	1	NIAGo4Life
NIH/NCCAM	National Center for Complementary & Alternative Medicine	1	NCCAM
NIH/NCI	National Cancer Institute	13	SmokefreeGov, NCIHINTS, etc.
NIH/NEI	National Eye Institute	1	NEHEP
NIH/NHLBI	National Heart, Blood & Lung Institute	3	TheHeartTruth, nih_nhlbi, BreatheBetter
NIH/NIAAA	National Institute of Alcohol Abuse & Alcoholism	1	NIAAAnews
NIH/NIAID	National Institute of Allergy & Infectious Diseases	3	NIAIDNews, NIAIDCareers, NIAIDFunding
NIH/NIAIMS	National Institute of Arthritis & Musculoskeletal & Skin Diseases	1	NIH_NIAMS
NIH/NICRR	National Center for Research Resources	1	ncrr_nih_gov
NIH/NIDA	National Institute of Drug Abuse	1	NIDAnews
NIH/NIEHS	National Institute of Environmental Health Sciences	1	NIEHS
NIH/NIGMS	National Institute of General Medical Sciences	1	NIGMS
NIH/NIHGRI	National Human Genome Research Institute	1	DNAday
NIH/NIMH	National Institute of Mental Health	1	NIMHgov
NIH/NLM	National Library of Medicine	11	NLM_LHC, medlineplus, NCBI, etc.
OIG	Office of Inspector General	1	OIGatHHS
OS	Office of the Secretary	29	AIDSgov, bestbones4ever, BirdFluGov, etc.
SAMHSA	The Substance Abuse & Mental Health Services	2	samhsagov, distressline
**Grand Total**		130	

We collected a total of 164,104 tweets from the timelines of the 130 handles. A third of the tweets (53,556) had zero retweets, i.e., generated no observable engagement. Less than 1% (613) had more than 100 retweets (total  = 174,395, mean  = 284). The remaining two-thirds (109,935) of tweets fell between these ranges (total  = 826,052, mean  = 7.5). Table 3 shows summary details about tweets and retweets per agency.

**Table 3:** Number of tweets and retweets per agency.

Agency	Date first handle was created	# tweets	# of tweets with zero retweets	# tweets with at least 1 retweet	# retweets	# retweets per tweet	# retweets per non-zero retweeted tweet
ACF	9/7/2011	605	219 (36.2%)	386 (63.8%)	1924	3.18	4.98
AHRQ	6/5/2009	1475	415 (28.14%)	1060 (71.86%)	3432	2.33	3.24
CDC	7/24/2008	**37136**	**11063 (29.79%)**	26073 (70.21%)	278885	7.51	10.70
CMS	9/1/2009	5620	2132 (37.94%)	3488 (62.06%)	11023	1.96	3.16
FDA	12/11/2008	10574	3007 (28.44%)	7567 (71.56%)	75245	7.12	9.94
HRSA	6/1/2009	1241	332 (26.75%)	909 (73.25%)	5391	4.34	5.93
NIH	6/16/2008	15550	7446 (47.88%)	8104 (52.12%)	49666	3.19	6.13
NIH/NIA	10/18/2011	1891	629 (33.26%)	1262 (66.74%)	10556	5.58	8.36
NIH/NCCAM	8/20/2009	1489	568 (38.15%)	921 (61.85%)	4102	2.75	4.45
NIH/NCI	4/28/2009	15679	5580 (35.59%)	10099 (64.41%)	46586	2.97	4.61
NIH/NEI	3/23/2011	401	249 (62.09%)	152 (37.91%)	331	0.83	2.18
NIH/NHLBI	2/26/2009	5135	1526 (29.72%)	3609 (70.28%)	29447	5.73	8.16
NIH/NIAAA	7/15/2010	424	122 (28.77%)	302 (71.23%)	2279	5.38	7.55
NIH/NIAID	7/24/2009	1725	830 (48.12%)	895 (51.88%)	2808	1.63	3.14
NIH/NIAIMS	8/31/2009	822	135 (16.42%)	687 (83.58%)	1850	2.25	2.69
NIH/NICRR	8/14/2009	1029	704 (68.42%)	325 (31.58%)	515	0.50	1.58
NIH/NIDA	1/5/2010	2191	669 (30.53%)	1522 (69.47%)	7484	3.42	4.92
NIH/NIEHS	12/17/2009	682	320 (46.92%)	362 (53.08%)	858	1.26	2.37
NIH/NIGMS	9/2/2009	983	420 (42.73%)	563 (57.27%)	1791	1.82	3.18
NIH/NIHGRI	2/25/2009	401	180 (44.89%)	221 (55.11%)	652	1.63	2.95
NIH/NIMH	5/11/2009	959	177 (18.46%)	782 (81.54%)	16779	17.50	21.46
NIH/NLM	2/12/2009	15058	6525 (43.33%)	8533 (56.67%)	48497	3.22	5.68
OIG	5/2/2011	1476	386 (26.15%)	1090 (73.85%)	2459	1.67	2.26
OS	**5/30/2007**	36587	8026 (21.94%)	**28561 (78.06%)**	**376158**	10.28	13.17
SAMHSA	3/17/2009	4971	1896 (38.14%)	3075 (61.86%)	21729	4.37	7.07
**Total**		164104	53556 (32.64%)	110548 (67.36%)	1000447	6.10	9.05
**Mean (SD)**		6564.16 (10355.66)	2142.24 (3055.12)	4421.92 (7499.23)	40017.88 (89880.72)	4.10 (3.64)	5.99 (4.39)

Bolded values indicate the largest values for the column.

In raw numbers we note that while the CDC posted the most tweets (37,136), it also has the highest raw number of tweets that are not retweeted (11,063). In contrast, the Office of the Secretary (OS), a close second in the number of total tweets (36,587), has the highest number of retweeted tweets (28,561) and also the highest number of retweets (376,158). Each tweet from OS gets approximately 10 retweets. The agency with the most retweets per retweeted tweet is NIH/NIMH with about 18 retweets per tweet. Also, it leads the agencies with 82% of its tweets retweeted at least once. Interestingly, this agency has less than 1000 tweets. Table 4 shows the top 10 handles ranked by the number of retweets per tweet. These are: CDCemergency (CDC), FitnessGov (OS), womenshealth (OS), HealthCareGov (OS), HHSGov (OS), FDArecalls (FDA), CDCgov (CDC), CDC_eHealth (CDC), NIMHgov (NIH/NIMH), and PHEgov (OS).

**Table 4:** Top 10 agency handles for most retweets per tweet.

Handle	Date of creation	# tweets	# of tweets with non-zero retweets	# of tweets with zero retweets	# retweets	# retweets per non-zero retweeted tweet
CDCemergency	1/28/2009	792	523 (66.04%)	269 (33.96%)	36756	**70.28**
FitnessGov	9/15/2011	935	834 (89.2%)	101 (10.8%)	23003	27.58
womenshealth	5/30/2007	**3236**	**3163 (97.74%)**	73 (2.26%)	**85832**	27.14
HealthCareGov	11/1/2009	409	404 (98.78%)	5 (1.22%)	10315	25.53
HHSGov	6/4/2009	1295	1103 (85.17%)	192 (14.83%)	26313	23.86
FDArecalls	12/11/2008	2118	1278 (60.34%)	**840 (39.66%)**	29764	23.29
CDCgov	5/21/2010	3226	2904 (90.02%)	322 (9.98%)	66204	22.80
CDC_eHealth	7/24/2008	1517	1255 (82.73%)	262 (17.27%)	27856	22.20
NIMHgov	5/11/2009	959	782 (81.54%)	177 (18.46%)	16779	21.46
PHEgov	4/26/2010	1356	998 (73.6%)	358 (26.4%)	20683	20.72

Bolded values indicate the largest values for the column.

88.46% of the retweeted tweets get their first retweet on the day of the tweet (referred to as day zero in our discussion). 60.6% of the retweeted tweets get their last retweet on day zero. Very few tweets receive their first tweet after 100 days. Similarly very few tweets get their last retweet after day 500.

**Figure 1:**
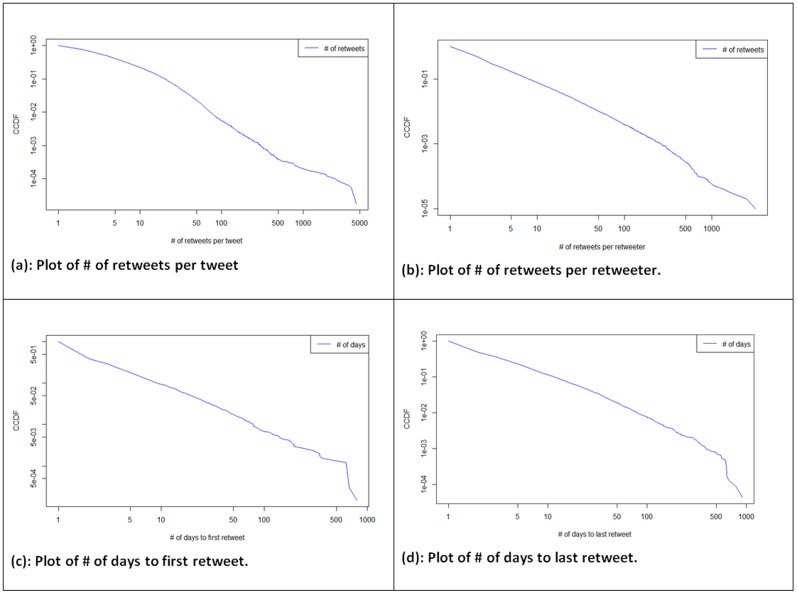
Power law plots of (a) retweets/tweet, (b) #retweets/retweeter, (c) #days to first retweet and (d) #days to last retweet.

We also study the power-law characteristics of different aspects of retweeting. With the exception of time to first retweet (power exponent  = 1.87), we find retweets/tweet (exponent  = 2.56), retweets/retweeter (exponent  = 2.35) and time to last retweet (exponent  = 2.33) have exponents in the range expected for power law distributions (between 2 and 3, with few exceptions). Concerning retweets/retweeter, we note that a few Twitter users retweet extensively (more than 500 times) while the majority of them retweet sparingly. Figure 1 shows these plots.

Concerning agencies, we find that 117 of the 130 HHS handles retweet each other's tweets. The top retweeting agencies are womenshealth with 2500 retweets followed by the NIH/NCI with 1662 retweets. MedicareGov, NCITechTransfer, NEHEP, NIAIDFunding and NIOSHManuf have the lowest retweet counts with 1 retweet each. Apart from these HHS handles, OrleansCoHealth, the Twitter handle of Orleans County Health Department (New York), has the highest retweeting activity with 3154 retweets.

### Tweet and Handle Features

**Figure 2:**
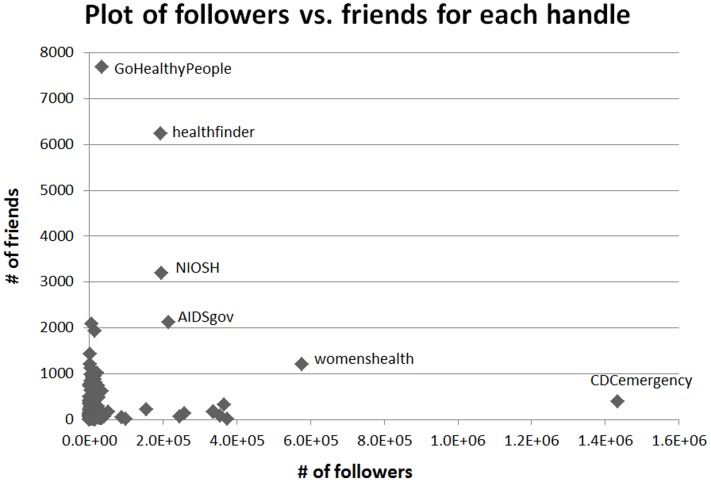
Plot of # of followers vs. # of friends for each handle. Few handles with disparate distribution of followers and friends have been labeled.

Figure 2 shows a scatter plot of followers versus friends. We find that CDCemergency has the highest number of followers (1,432,424) but very few friends (393). On the other hand GoHealthyPeople has many friends (7,688) but few followers (34,913). NIAIDCareers (1008: 729) and distressline (1701: 1203) have relatively balanced number of followers and friends in comparison to the overall ratio of followers and friends for the different handles (49832: 405).

NLM_DIMRC has the highest number (575) of favorites, followed by GoHealthyPeople (343) and AIDSgov (216). 50 handles (e.g. NIHLBI, DNADay, NCBI) did not have any favorites.

The top ranking handles in tweet count are CDCSTD (12151), womenshealth (9419), CDCNPIN (9157), NIOSH (8936) and talkHIV (7663) and the lowest 5 are ncbi_pubmed (60), NCISymptomMgmt (144), NIOSH_FirRanges (150), FDACBER (162), and Medicare_Fraud (171).

NIHforHealth, CDCgov and HHSGov have the highest betweenness-centrality values of 987.2, 851.51 and 717.54 respectively. Betweenness-centality does not apply to NIHforFunding and nlm_newsroom as these are nodes with zero in- or out-degrees.

An overwhelming portion, 75% of tweets in our dataset contain URLs. Around 57% contain hashtags while 38% contain user-mentions.

Table 5 shows the distribution of tweets across sentiment scales. We find that in general slightly more tweets are classified as negative (percentage of moderate to extreme negative is 32.2% while for positive this percentage is 28.3%).

**Table 5:** Distribution of positive and negative sentiments for tweets on a 5-point scale.

	# of positive tweets	# of negative tweets
neutral	117599 (71.66%)	111233 (67.78%)
moderate-medium	36940 (22.51%)	31791 (19.37%)
medium	8502 (5.18%)	10143 (6.18%)
medium-extreme	1051 (0.64%)	10772 (6.56%)
extreme	12 (0.01%)	165 (0.10%)
**Total**	164104	164104

Table 6 shows the 15 semantic groups with examples of component semantic types and their prevalence in our dataset. “Concepts & Ideas” (41.68% tweets) is the most prevalent group followed by “Disorders” and “Living Beings” (around 36% for each). “Genes & Molecular Sequences” is least frequent (0.69%). Health agencies more often discuss concepts and ideas or disorders than amino acid and carbohydrate sequences.

**Table 6:** Semantic groups with examples of component semantic types and their prevalence in the dataset.

Semantic Groups	Example Semantic Types	# of tweets (%)
Concepts & Ideas	Functional Concept, Regulation or Law, Temporal Concept, etc.	68391 (41.68%)
Disorders	Anatomical Abnormality, Disease or Syndrome, Neoplastic Process, etc.	59164 (36.05%)
Living Beings	Mammal, Eukaryote, Plant, etc.	57836 (35.24%)
Geographic Areas	Geographic Area	42133 (25.67%)
Chemicals & Drugs	Clinical Drug, Organic Chemical, Enzyme, etc.	39065 (23.81%)
Activities & Behaviors	Daily or Recreational Activity, Machine Activity, Social Behavior, etc.	38276 (23.32%)
Organizations	Health Care Related Organization, Professional Society, Self-help or Relief Organization	35163 (21.43%)
Physiology	Cell Function, Mental Process, Organ or Tissue Function, etc.	32308 (19.69%)
Objects	Entity, Food, Manufactured Object, etc.	23452 (14.29%)
Procedures	Diagnostic Procedure, Research Activity, Therapeutic or Preventive Procedure, etc.	23445 (14.29%)
Phenomena	Biologic Function, Human-caused Phenomenon or Process, Natural Phenomenon or Process	20252 (12.34%)
Anatomy	Anatomical Structure, Cell Component, Tissue, etc.	7925 (4.83%)
Occupations	Biomedical Occupation or Discipline, Occupation or Discipline	7633 (4.65%)
Devices	Drug Delivery Device, Medical Device, Research Device	1610 (0.98%)
Genes & Molecular Sequences	Amino Acid Sequence, Carbohydrate Sequence, Gene or Genome, etc.	1138 (0.69%)

We also compared the tweets posted by the health agencies with news in traditional media. The influence of traditional news sources on social media has been studied [43]–[45] but not in health. Google Health News is an aggregator that has been shown to be useful in infectious disease monitoring [46]. Gathering news from it we find surprisingly little overlap with agency tweets. Only 1601 tweets (<1% of the total) overlap with news headlines. Of these, tweets and news appear on the same day in 320 cases, tweets precede news in 610 cases and news precedes tweet in 671 cases. Our results with health agency tweets is consistent with previous studies finding topics discussed in Twitter to be considerably different from traditional news sources [43].

### Hurdle Model Analysis of Tweets

Results from the hurdle model are given in Table 7. But first, an important assumption in multiple regression analysis is that the variables used in the statistical models are independent of each other i.e. multicollinearity should not exist among them. We use the variance inflation factor (VIF) to check for the presence of multicollinearity in our experiments. VIF scores for all independent variables in our regression analysis were within the range of zero to 5 indicating no multicollinearity issues.

**Table 7:** Results of hurdle negative binomial model showing the estimate/coefficient (SE), exponent of coefficient (OR and IRR), z and p-values (*p<0.05, **p<0.01, ***p<0.001) for various independent variables for zero and count portions of the model.

	Zero Portion				Count Portion			
	Estimate (SE)	OR	z value	p	Estimate (SE)	IRR	z value	p
(Intercept)	−3.295 (0.05)	0.037	−65.69	***	−1.361 (0.053)	0.256	−25.89	***
Log-transformed Favorite Count	**0.207 (0.009)**	**1.23**	**23.668**	***	**0.074 (0.009)**	**1.077**	**8.025**	***
Log-transformed Follower Count	**0.922 (0.012)**	**2.515**	**74.148**	***	**0.939 (0.011)**	**2.559**	**85.831**	***
Log-transformed Friend Count	0.002 (0.012)	1.002	0.168		*−0.181 (0.013)*	*0.835*	*−13.717*	***
Log-transformed Tweet Count	*−1.242 (0.02)*	*0.289*	*−61.38*	***	*−0.712 (0.019)*	*0.491*	*−37.481*	***
Log-transformed betweenness-centrality	0.016 (0.01)	1.016	1.679		**0.099 (0.01)**	**1.105**	**10.347**	***
Log-transformed tweet age	**1.108 (0.016)**	**3.029**	**69.973**	***	**0.12 (0.018)**	**1.128**	**6.539**	***
Hashtag	**0.386 (0.012)**	**1.471**	**32.662**	***	*−0.034 (0.011)*	*0.966*	*−3.01*	**
URL	**0.529 (0.014)**	**1.697**	**38.084**	***	*−0.08 (0.014)*	*0.923*	*−5.581*	***
User-mention	**0.229 (0.012)**	**1.257**	**18.355**	***	**0.869 (0.012)**	**2.385**	**72.131**	***
Positive Sentiment	*−0.08 (0.009)*	*0.923*	*−8.473*	***	−0.016 (0.01)	0.984	−1.695	
Negative Sentiment	*−0.141 (0.008)*	*0.868*	*−18.747*	***	*−0.056 (0.007)*	*0.945*	*−8.222*	***
Activities & Behaviors	**0.32 (0.014)**	**1.377**	**22.869**	***	**0.175 (0.013)**	**1.191**	**13.213**	***
Anatomy	**0.195 (0.028)**	**1.215**	**6.959**	***	*−0.05 (0.025)*	*0.951*	*−2.026*	*
Chemicals & Drugs	**0.105 (0.014)**	**1.11**	**7.675**	***	**0.141 (0.013)**	**1.151**	**10.82**	***
Concepts & Ideas	**0.235 (0.012)**	**1.265**	**19.933**	***	−0.022 (0.011)	0.978	−1.94	
Devices	**0.273 (0.059)**	**1.314**	**4.653**	***	*−0.226 (0.054)*	*0.797*	*−4.224*	***
Disorders	**0.278 (0.014)**	**1.32**	**20.516**	***	**0.177 (0.013)**	**1.193**	**13.909**	***
Genes & Molecular Sequences	0.058 (0.071)	1.059	0.812		*−0.952 (0.065)*	*0.386*	*−14.674*	***
Geographic Areas	*−0.037 (0.018)*	*0.964*	*−1.986*	*	*−0.324 (0.018)*	*0.723*	*−18.216*	***
Living Beings	**0.083 (0.012)**	**1.086**	**6.643**	***	**0.082 (0.012)**	**1.085**	**6.927**	***
Objects	**0.14 (0.017)**	**1.15**	**8.331**	***	**0.192 (0.016)**	**1.212**	**11.979**	***
Occupations	*−0.057 (0.027)*	*0.945*	*−2.146*	*	*−0.134 (0.027)*	*0.875*	*−5*	***
Organizations	*−0.107 (0.02)*	*0.899*	*−5.394*	***	*−0.24 (0.019)*	*0.786*	*−12.683*	***
Phenomena	**0.07 (0.018)**	**1.073**	**3.939**	***	**0.436 (0.017)**	**1.547**	**25.043**	***
Physiology	**0.188 (0.015)**	**1.207**	**12.465**	***	**0.311 (0.014)**	**1.365**	**22.106**	***
Procedures	**0.046 (0.017)**	**1.047**	**2.733**	**	*−0.082 (0.016)*	*0.921*	*−5.157*	***
Log(theta)					−1.8 (0.024)	0.165	−73.547	***

Italicized rows: variables with significant negative association, bolded rows: variables with significant positive association. The Zero Portion is a model of whether or not there is a retweet and the Count Portion models the number of retweets.

For the zero portion of the hurdle model – modeling whether a retweet occurs or not – increases in the number of favorites and followers are positively associated with retweets, as is tweet age. Tweet count, however, is negatively associated with retweets. Hashtags, URLs and user-mentions – are positively associated with retweets. Both positive and negative sentiments are associated with a lower probability of retweeting. Almost all semantic groups, except for geographic areas, occupations and organizations, are positively associated with retweeting.

For the count portion of the hurdle model – modeling the number of retweets – the results are similar to those of the zero portion with a few exceptions: friend count, which was insignificant in the zero portion, is negatively associated with number of retweets. Hashtags and URLS are negatively associated with the number of retweets. Also, some semantic groups are negatively associated with retweet counts, but positively associated with whether or not a retweet occurred, specifically anatomy, devices, genes & molecular sequences and procedures.

### Cox Models of Tweets

We estimated two Cox proportional hazards models. First, we modeled time to first retweet, and the results are presented in Table 8. In this case, shorter time periods are preferred. Time to retweet is shorter for handles that have more favorites and followers. It is also shorter for tweets with longer tweet age and the presence of hashtags. Time to retweet is longer for increases in friend count, user-mentions, and positive sentiment. Most of the semantic groups are not associated with time to first retweet.

**Table 8:** Results of Cox proportional hazards model for interval between a tweet and its first retweet.

	Interval Between Tweet and First Retweet			
	Coefficient (SE)	HR	z	p
Log-transformed Favorite Count	**0.055 (0.006)**	**1.056**	**8.72**	***
Log-transformed Follower Count	**0.102 (0.009)**	**1.107**	**11.029**	***
Log-transformed Friend Count	*−0.026 (0.009)*	*0.974*	*−2.929*	**
Log-transformed betweenness	−0.004 (0.008)	0.995	−0.566	
Log-transformed Tweet Count	0.017 (0.015)	1.017	1.176	
Log-transformed tweet age	**0.089 (0.012)**	**1.093**	**7.204**	***
Hashtag	**0.116 (0.009)**	**1.123**	**12.873**	***
URL	−0.021 (0.011)	0.978	−1.907	.
User-mention	*−0.072 (0.01)*	*0.930*	*−7.186*	***
Positive Sentiment	*−0.02 (0.007)*	*0.979*	*−2.807*	**
Negative Sentiment	−0.001 (0.005)	0.998	−0.284	
Activities & Behaviors	0.008 (0.01)	1.008	0.827	
Anatomy	0.001 (0.019)	1.001	0.077	
Chemicals & Drugs	0.013 (0.01)	1.013	1.347	
Concepts & Ideas	0.008 (0.009)	1.008	0.954	
Devices	−0.009 (0.04)	0.990	−0.231	
Disorders	**0.02 (0.01)**	**1.020**	**2.037**	*
Genes & Molecular Sequences	0.051 (0.047)	1.052	1.085	
Geographic Areas	*−0.033 (0.014)*	*0.967*	*−2.296*	*
Living Beings	0.006 (0.009)	1.006	0.667	
Objects	0 (0.012)	0.999	−0.033	
Occupations	−0.02 (0.02)	0.980	−0.998	
Organizations	0.006 (0.015)	1.006	0.416	
Phenomena	0.021 (0.013)	1.021	1.596	
Physiology	0.009 (0.011)	1.009	0.827	
Procedures	**0.024 (0.012)**	**1.024**	**2.006**	*

The Coefficients (SE), hazard ratios (HR), z and p-values (*p<0.05, **p<0.01, ***p<0.001) for the independent variables are shown. Italicized rows: variables with significant negative association, bolded rows: variables with significant positive association.

Second, we modeled the time to the last retweet, and the results are presented in Table 9. In this case, longer time periods are preferred. Longer time to the last retweet is associated with the handle's follower count, the presence of a URL in the tweet, and positive sentiment. Handles with more favorites, higher tweet count, and increased betweenness-centrality, as well as tweets with user-mention, hashtags and negative sentiment have shorter times to last retweet.

**Table 9:** Results of Cox proportional hazards model for interval between a tweet and its last retweet.

	Interval Between Tweet and Last Retweet			
	Coefficient (SE)	HR	z	p
Log-transformed Favorite Count	**0.037 (0.006)**	**1.037**	**5.912**	***
Log-transformed Follower Count	*−0.27 (0.009)*	*0.763*	*−29.249*	***
Log-transformed Friend Count	−0.009 (0.009)	0.991	−0.972	
Log-transformed Tweet Count	**0.351 (0.015)**	**1.420**	**23.994**	***
Log-transformed tweet age	−0.014 (0.012)	0.986	−1.124	
Log-transformed betweenness	**0.036 (0.008)**	**1.036**	**4.832**	***
Hashtag	**0.139 (0.009)**	**1.149**	**15.519**	***
URL	*−0.179 (0.011)*	*0.835*	*−15.915*	***
User-mention	**0.094 (0.01)**	**1.098**	**9.355**	***
Positive Sentiment	*−0.025 (0.007)*	*0.975*	*−3.411*	***
Negative Sentiment	**0.037 (0.005)**	**1.037**	**7.049**	***
Activities & Behaviors	*−0.043 (0.01)*	*0.957*	*−4.265*	***
Anatomy	*−0.038 (0.019)*	*0.962*	*−1.961*	*
Chemicals & Drugs	−0.019 (0.01)	0.981	−1.936	
Concepts & Ideas	−0.011 (0.009)	0.989	−1.262	
Devices	−0.059 (0.04)	0.942	−1.471	
Disorders	−0.011 (0.01)	0.988	−1.156	
Genes & Molecular Sequences	0.059 (0.047)	1.060	1.252	
Geographic Areas	0.001 (0.014)	1.000	0.04	
Living Beings	−0.006 (0.009)	0.993	−0.721	
Objects	*−0.049 (0.012)*	*0.951*	*−3.993*	***
Occupations	**0.04 (0.02)**	**1.040**	**1.977**	*
Organizations	**0.041 (0.015)**	**1.041**	**2.687**	**
Phenomena	−0.012 (0.013)	0.987	−0.928	
Physiology	−0.013 (0.011)	0.986	−1.189	
Procedures	0.017 (0.012)	1.016	1.388	

The Coefficients (SE), hazard ratios (HR), z and p-values (*p<0.05, **p<0.01, ***p<0.001) for the independent variables are shown. Italicized rows: variables with significant negative association, bolded rows: variables with significant positive association.

## Discussion

Our results show that although multiple federal health agencies are using Twitter, there is a great deal of difference between levels of Twitter use and also retweets. For public health agencies, we found that a tiny minority of tweets gets more than 100 retweets; a two-thirds majority of tweets get on average 8 retweets. We also found that a handle's follower count and favorite count have strong positive relationships with retweeting behavior. While these features are not easy for agencies to improve, they are easy metrics to follow. In contrast, we found that having more friends on Twitter was negatively associated with the number of times a tweet is retweeted.

Early adoption of Twitter by an agency is associated with our measures of engagement. As a handle ages the chances for engagement overall seem to improve. This is consistent with findings in the general Twitter domain [47]. This is not something that agencies can change but it does provide support for health agencies thinking about starting Twitter accounts to do just that and not to wait and delay getting started.

Agencies generating more tweets than others do not necessarily have more retweets. In fact, we found that tweet count, the number of tweets posted overall, is negatively associated with retweets. This is consistent with anecdotal evidence from the web [48], [49]. This suggests that an agency might consider only tweeting posts that it regards as important so as to not ‘dilute’ the public's attention. However, this observation must be balanced against the fact that information dissemination on a topic may be an organization's main goal and not necessarily public response. In that case regular or even frequent postings related to a message may be appropriate.

Health agencies can augment their tweets by adding hashtags, URLs, or user-mentions and this may increase the likelihood that users will find the information encoded in the tweet more useful and thus retweet it. Indeed, we found that the addition of hashtags, URLs, or user-mentions did indeed increase the likelihood that a given tweet would be retweeted. However, the inclusion of hashtags and URLs is also associated with decreased numbers of retweets, and user-mentions are associated with shorter times to last retweet. Thus, agencies may be able to increase retweets by using these conventions, but they might not increase the longevity of tweets. Our user-mentions results are in slight contrast to previous research, which found these to have (marginally significant) *negative* associations with retweeting [47]. But our results for hashtags and URLs are generally consistent with previous results [47], [50].

Our observations regarding hashtags, user-mentions and URLs are also interesting because of differences in their prevalence between our dataset and Twitter data in general. The agency tweets in this paper use more URLs than found in the general domain, 75% vs. 19% [51] and 21% [47]. We speculate that this abundance of URLs for tweets from health agencies may be because in health communications references to sources and supporting materials are necessary. This is supported by another study on the use of Twitter by local health departments where the authors found 74% of tweets contain URLs [52]. Hashtags and user-mentions are also more prevalent in our dataset appearing in 57% and 38% of agency tweets respectively, while in the general domain hashtags were found in only 16% and user-mentions in only 20% of tweets [52].

Betweenness-centrality is positively related to the number of retweets and negatively related to the time to last retweet. While betweenness-centrality has been used extensively in social media research in various domains ranging from health to politics [53]–[56], in most cases it is used as a metric of influence in a retweet or a reply network. To the best of our knowledge, researchers have not explored the direct association of betweenness centrality scores to retweeting activity. We speculate that since we calculated betweenness-centrality based on the follower-following network among agencies, an agency with high betweenness-centrality, i.e. following many other federal agencies, may not have any major effect on the rate or lifespan of retweets.

Much work has been done involving mining sentiment from Twitter and it has previously been demonstrated that the presence of sentiment of one kind or the other is associated with higher rates of retweeting [57]–[59]. In contrast, we found that sentiment in tweets from government agencies, either positive or negative, is not associated with retweeting. It should also be noted that agency tweets are predominantly neutral (70%).

Semantic groups have not been studied in the context of retweet rates. We found that posts about activities and behaviors, chemicals and drugs, disorders, living beings, objects, phenomenon and physiology are positively associated with engagement. In contrast, posts about organizations, occupations, genes & sequences and geographic areas tend to lower engagement. But it may also be that the intent behind such posts are less to engage and more to just inform.

## Limitations

Our study has a few limitations. First, it is comprised of observational data; i.e., we did not run formal experiments. Thus although we can describe associations, we cannot establish causality. For example, while we find that the number of followers is associated with retweeting, we cannot insure, due to the descriptive nature of the study, that increasing the number of followers will lead to an increase in retweets. Second, although we captured the majority of tweets from federal agencies we could only collect a maximum of 3200 for each handle, so for a few of these agency handles (18/130), our data was censored. Nevertheless, we still had a large corpus of tweets over a long period of time. Third, the intent behind some tweets may simply be to inform and not necessarily to engage via retweeting. We do not know about an organization's motivations for tweeting or for posting specific tweets or the targeted audience. Furthermore, some agencies may have more information that naturally draws the public. Thus, these results do not represent a “report card” on these agencies. Fourth, our definition of engagement is limited to examining retweeting and its features. Fifth, although we considered various important and typically used tweet-based features in our statistical analysis, there may be other key features. For example, while time or day of the week may have significant effects on tweeting or retweeting behavior [60], [61] and hence engagement, these features were considered outside the scope of our study. We also did not examine the features of the retweet. For example, a retweet may agree with or contradict the message in the source tweet. Finally we limit our analysis to Twitter, and there are other social network platforms that federal agencies are using.

## Conclusions

We present the first comprehensive analyses of Twitter engagement by public health agencies. The level of Twitter activity varies greatly by health agency: some health accounts are very active and others are not as much. However, it seems to be the content of the Tweets (e.g., about activities and behaviors, disorders) and not the number of tweets alone that is associated with a higher level of engagement (number of retweets). Furthermore, although some of the factors associated with more engagement cannot be changed by the agency (e.g., the length of time they have been active on Twitter), several factors associated with higher retweets can be controlled (e.g., use of hashtags, URLs). Our results provide a framework for future experiments designed to improve the public's engagement with health agencies via Twitter.

## Supporting Information

Data S1 **List of Twitter handles of 130 HHS health agencies used in this paper.**
(TXT)
